# Hepatic artery intervention combined with immune-targeted therapy is superior to sequential therapy in BCLC-C hepatocellular carcinoma

**DOI:** 10.1007/s00432-022-04386-3

**Published:** 2022-12-01

**Authors:** Hanzhi Dong, Yan Jian, Meijian Wang, Fangfang Liu, Qi Zhang, Zhiqiang Peng, Na Cheng, Wenfeng Zhang

**Affiliations:** 1 Department of Medical Oncology, Jiangxi Cancer Hospital, The Second Affiliated Hospital of Nanchang Medical College, Jiangxi Clinical Research Center for Cancer, Nanchang, 330029 China; 2 The First Affiliated Hospital, Nanchang University, Nanchang, 330006 Jiangxi China; 3 Department of Liver Disease Center, Shenzhen Hospital of Southern Medical University, Shenzhen, 518000 Guangdong China; 4 Department of Lymphohematology, Jiangxi Cancer Hospital, The Second Affiliated Hospital of Nanchang Medical College, Jiangxi Clinical Research Center for Cancer, Nanchang, 330029 China; 5 Department of Infectious Diseases, The First Affiliated Hospital, Nanchang University, Nanchang, 330006 Jiangxi China

**Keywords:** Hepatic artery intervention, Target therapy, PD-1 inhibitors, BCLC-C hepatocellular carcinoma

## Abstract

**Background:**

Hepatic artery intervention combined with immunotarget therapy exerts excellent disease control and prolongs survival. However, the arrangement of hepatic artery intervention and systemic therapy confuses clinical decisions.

**Methods:**

A two-center, retrospective clinical study was approved by the Institutional Ethics Committee. From December 2018 to February 2022, patients with Barcelona Clinic Liver Cancer stage C (BCLC-C) hepatocellular carcinoma (HCC) who received targeted therapy plus PD-1 inhibitors with or without hepatic artery intervention were included. According to the treatment mode, the patients were assigned to three groups: initial hepatic artery intervention combined with immunotarget therapy, immunotarget therapy sequential hepatic artery interventional therapy, and immunotarget therapy only. The survival, response, and adverse events were compared among the three groups. Subgroup analysis and univariate and multivariate prognostic analyses were also evaluated.

**Results:**

The median follow-up time was 18.3 months (95% CI 16.7 to 20.0 months). A total of 163 patients with BCLC-C stage HCC were assigned to three groups: initial hepatic artery intervention plus PD-1 inhibitors plus targeted therapy (HPT, *n* = 66), PD-1 inhibitors plus targeted therapy followed by hepatic artery intervention (PTH, *n* = 56) and PD-1 inhibitors plus targeted therapy (PT, *n* = 41). The median progression-free survival was 8.37 months (95% CI 6.35–10.39) with HPT versus 5.3 months (95% CI 3.48–7.12) with PTH versus 6.33 months (95% CI 3.75–8.92) with PT. The progression-free survival of the HPT group was better than that of the PTH group (HR 0.66, 95% CI 0.45–0.97, *p* = 0.027) and PT group (HR 0.60, 95% CI 0.39–0.92, *p* = 0.01). The median overall survival was 14.6 months (95% CI 10.6–18.7) with HPT, 10.0 months (95% CI 8.2–11.8) with PTH and 11.3 months (95% CI 8.3–14.3) with PT. The 1-year overall survival (OS) rates in the HPT, PTH and PT groups were 50%, 33.9%, and 34.1%, respectively. Overall survival was significantly longer in the HTP group than in the PT group (HR 0.60, 95% CI 0.361–0.996, *p* = 0.032). Compared with the PTH group, the overall survival of the HTP group had a prolonged survival trend (HR 0.66, 95% CI 0.416–1.032, *p* = 0.059). All treatment modalities were deemed equally safe. Multivariate analysis suggested that the mode of treatment, albumin level, Child‒Pugh grade and hepatectomy history were independent prognostic factors for BCLC-C HCC patients.

**Conclusions:**

Initial hepatic artery intervention combined with immunotarget therapy gained survival benefits with tolerable side effects compared with immunotarget sequential hepatic artery intervention and immunotarget therapy alone. Multivariate analysis suggested that liver reserve function was closely correlated with prognosis.

**Supplementary Information:**

The online version contains supplementary material available at 10.1007/s00432-022-04386-3.

## Introduction

Primary liver cancer is the second leading cause of oncological mortality and the seventh most commonly diagnosed cancer worldwide according to the International Agency for Research on Cancer in 2020 (Sung et al. 2021). Hepatocellular carcinoma (HCC) has diverse genetic mutations and multiple signaling pathways and is a highly heterogeneous tumor susceptible to therapy resistance (Llovet et al. 2018). Patients with early and intermediate-stage disease can benefit from local treatments, such as surgery, hepatic artery intervention therapy, or local ablation, as reported in previous studies, with a median overall survival of more than 25 months (Llovet et al. 2021a, b) whereas advanced HCC (Barcelona Clinic Liver Cancer stage C, BCLC-C) has a less favorable prognosis, and the median overall survival achieved with systemic treatment is approximately 1 year (Li et al. 2021a).

BCLC stage C hepatocellular carcinoma is characterized by vascular invasion or extrahepatic metastasis with Child‒Pugh grade A or B. Sorafenib, lenvatinib, pembrolizumab, durvalumab, and atezolizumab plus bevacizumab are the first-line, standardized treatments for advanced hepatocellular carcinoma (Vogel and Martinelli 2021). The survival benefit of systematic treatment is limited, and other treatment options, including transcatheter arterial chemoembolization (TACE) and hepatic arterial infusion chemotherapy (HAIC), are also widely used clinically. Some studies suggested that sorafenib plus HAIC was associated with longer survival than sorafenib or HAIC alone (He et al. 2019; Regmi et al. 2021). However, the SCOOP-2 trial reported that sequential HAIC with cisplatin and sorafenib did not improve the survival benefit compared with sorafenib alone (Kondo et al. 2019). Therefore, whether hepatic artery interventional therapy plus targeted therapy should be used is controversial.

PD-1 inhibitors combined with targeted therapy are a promising treatment for advanced HCC and can prolong OS for some patients (Finn et al. 2020; Yau et al. 2022; Kudo 2022). Nevertheless, it is unclear whether immunotarget therapy plus hepatic artery interventional therapy is superior to immunotarget therapy alone. In addition, the optimal placement order of hepatic artery interventional therapy and immunotarget therapy remains unclear.

Herein, we aimed to further explore the optimal treatment mode for patients with BCLC stage C HCC. A total of 163 patients with BCLC stage C HCC were screened from two centers, and retrospective analysis was performed to compare the efficacy and safety of three treatment modes: immunotarget therapy alone, initial hepatic artery intervention combined with immunotarget therapy, and immunotarget therapy sequential hepatic artery interventional therapy. In addition, univariate and multivariate analyses were used to identify the best beneficiaries.

## Materials and methods

### Study design and patients

This was a dual-center, retrospective, observational study according to the ethical guidelines of the 1975 Declaration of Helsinki. The collection and analysis of patient data were approved by the Human Ethics Committee at the First Affiliated Hospital of Nanchang University and Jiangxi Cancer Hospital (Jiangxi, China).

### Patients

In the present study, a total of 163 patients were included: 66 patients treated with initial hepatic artery intervention combined with immunotarget therapy, 56 patients treated with immunotarget therapy followed by hepatic artery intervention therapy, and 41 patients treated with immunotarget therapy only. The participants were derived from two clinic centers and were enrolled between December 2018 and February 2022. The inclusion criteria included the following: all patients were confirmed as having HCC by pathology or the American Association for the Study of Liver Disease criteria, at least 18 years old, no sex limitation, Barcelona Clinic Liver Cancer stage C (BCLC-C), Eastern Cooperative Oncology Group (ECOG) Performance Status score 0–2, at least one measurable lesion according to Modified Response Evaluation Criteria in Solid Tumors (mRECIST) (Llovet and Lencioni 2020), Child‒Pugh A to B7, no other malignant tumors, no other antitumor treatments combination, at least 2 treatment cycles, complete medical records, and follow-up. The exclusion criteria included prior immunotherapy, autoimmune disease, or inflammatory disorders.

### Treatment procedure

All patients were assigned to three groups based on treatment as follows: hepatic artery intervention plus immunotarget therapy within 21 days was defined as initial hepatic artery intervention combined with immunotarget therapy, abbreviated as HPT; after 21 days of immunotarget therapy followed by hepatic artery interventional therapy was defined as immunotarget therapy sequential hepatic artery interventional therapy, abbreviated as PTH; and immunotarget therapy, abbreviated as PT.

Hepatic artery interventional therapy included HAIC and TACE. Systemic therapy included targeted therapy and PD-1 inhibitors. (Table S1).

### HAIC procedure

HAIC treatment was divided into 3-week cycles. The microcatheter was advanced into the hepatic artery according to a previously reported protocol on day 1 in every cycle of treatment, and patients were transferred to the inpatient ward for drug infusion via the hepatic artery as follows: oxaliplatin, 130 mg/m^2^ from hour 0–2 on day 1; leucovorin, 400 mg/m^2^ from hour 2–3 on day 1; and fluorouracil, 400 mg/m^2^ bolus at hour 3 on day 1 and 2400 mg/m^2^ over 24 h.

### TACE procedure

TACE was performed according to a previously reported protocol. A 3.5 French catheter was inserted into the celiac trunk or superior mesenteric artery for arteriography. Then, a 2.7 French microcatheter was superselectively placed into the feeding arteries of the tumors. Chemolipiodolization was performed using 50 mg of epirubicin and 50 mg of lobaplatin mixed with lipiodol. Subsequently, embolization was performed with the injection of polyvinyl alcohol particles. Repeated TACE cycles were performed every 5–6 weeks.

### Systemic therapy

Lenvatinib was taken orally at 8–12 mg according to patients’ body weights, and other targeted medicine and PD-1 inhibitors were administered in standard doses. (Table S1).

### Data collection

Patient information was collected from electronic medical records to ensure data authenticity and traceability at each center. Collected data included demographic characteristics (age, sex, height, and weight), disease characteristics (ECOG performance status, status of HBV infection, Child‒Pugh, liver cirrhosis status, tumor thrombus, and extrahepatic metastasis), medical history, laboratory examination (AFP, leukocyte count, hemoglobin, platelets, lymphocyte count, TBIL, ALT, AST, ALB, LDH, and PT) and imaging data [computed tomography (CT) or magnetic resonance imaging (MRI)]. Adverse events were recorded within 60 days after treatment. Adverse event classification was based on the National Cancer Institute’s Common Terminology Criteria version 5.0.

### Response assessment

Baseline imaging was performed within 1 month prior to treatment. Imaging follow-up was performed every 2–3 months with the assistance of two experienced radiologists to evaluate the response. Therapeutic evaluation was assessed according to the Modified Response Evaluation Criteria in Solid Tumors (mRECIST).

### Follow-up

Surveillance strategies were unified at both centers. Specifically, AFP and abdominal imaging were reviewed every 2–3 months after treatment. Abdominal imaging was defined as contrast-enhanced CT or MRI imaging whereas abdominal ultrasound was not used to evaluate the response. The dates of first treatment, disease progression, last follow-up, and death were recorded.

### Statistical analysis

The chi-square test or Fisher’s Exact test were used for categorical variables, and one-way ANOVA was used for continuous variables of baseline demographics and clinical characteristics. Categorical variables were described by frequencies or percentages, and continuous variables were described by medians and ranges. The progression-free survival (PFS) and overall survival (OS) were calculated with the Kaplan‒Meier method. Univariate and multivariable Cox regression analyses were used to identify the risk factors for PFS and OS. The hazard ratio (HR) and 95% confidence intervals (CI) were calculated. Subgroup analyses were performed in patients who received HPT and PT, and the dominant population in the two treatment models was analyzed. Statistical analyses were performed using SPSS version 28.0 (IBM) and GraphPad Prism version 8.0 (GraphPad, Inc.). A 2-tailed *P* value < 0.05 was considered statistically significant for all data analyses.

## Results

**Fig. 1:**
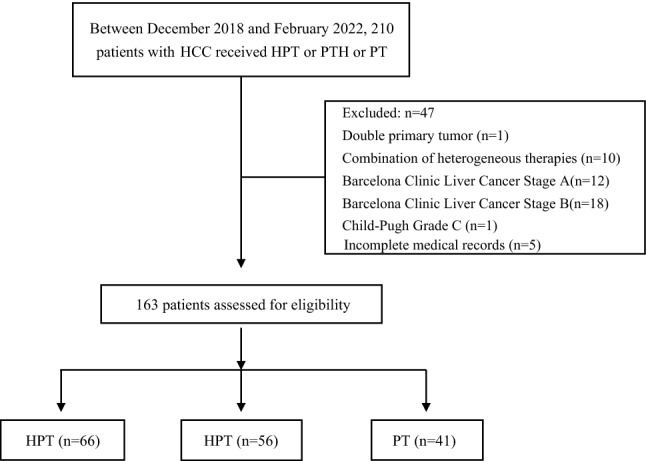
Patient flow chart

Between December 4, 2018, and February 3, 2022, 210 patients were screened, of whom 47 were excluded and 163 were enrolled. The 163 included patients with BCLC-C HCC were assigned to three groups: initial hepatic artery intervention plus PD-1 inhibitors plus targeted therapy (HPT, *n* = 66), PD-1 inhibitors plus targeted therapy followed by hepatic artery intervention (PTH, *n* = 56), and PD-1 inhibitors plus targeted therapy (PT, *n* = 41) (Fig. 1).

### Baseline demographics and clinical characteristics

The baseline demographics and clinical characteristics were well balanced among the three groups (Table 1). The mean age of patients in the PT group was slightly older than that in the HPT and PTH groups. Most patients were male (82.93–91.07%), but the percentages of males and females in each group were balanced. PS = 1 and Child‒Pugh A were dominant in each group, accounting for approximately 75–89%. The number of patients with AFP greater than 400 ng/ml in the PTH group and PT group was slightly higher than that in the HPT group. Other characteristics were similarly distributed in each group, such as laboratory tests, liver cirrhosis, tumor thrombus, extrahepatic metastasis, smoker status, alcoholism, and medical history.

**Table 1:** Baseline clinical characteristics of patients

Characteristic*	HPT (*n* = 66)	PTH (*n* = 56)	PT (*n* = 41)	*P* value
Age	52 (40–65)	52 (41–64)	57 (47–67)	0.074
*Gender*				0.416
Male	57 (86.36)	51 (91.07)	34 (82.93)	
Female	9 (13.64)	5 (8.93)	7 (17.07)	
*PS*				0.191
0	11 (16.67)	2 (3.57)	7 (17.10)	
1	50 (75.76)	50 (89.29)	31 (75.60)	
2	5 (7.58)	4 (7.14)	3 (7.30)	
BMI	21.64 ± 3.07	21.09 ± 2.87	22.36 ± 3.42	0.135
*HBV*				0.191
Negative	12 (18.18)	4 (7.14)	5 (12.20)	
Positive	54 (81.82)	52 (92.86)	36 (87.80)	
*Child–pugh*				0.995
A	50 (75.76)	42 (75.00)	31(75.60)	
B	16 (24.24)	14 (25.00)	10 (24.40)	
*AFP*				0.199
< 400	39 (59.09)	28 (50)	20 (48.78)	
≥ 400	27 (40.91)	28 (50)	21 (51.22)	
WBC	5.36 ± 2.20	5.83 ± 2.36	5.42 ± 2.08	0.475
HB	129.33 ± 21.99	130.16 ± 23.10	125.12 ± 22.53	0.519
PLT	149.90 ± 82.67	166.20 ± 88.09	162.39 ± 133.52	0.642
LYM	1.02 ± 0.52	1.13 ± 0.52	1.05 ± 0.46	0.516
TBIL	21.95 ± 14.53	22.06 ± 21.13	22.62 ± 22.83	0.984
ALT	71.43 ± 60.36	81.45 ± 65.30	68.03 ± 48.59	0.494
AST	48.36 ± 38.23	51.43 ± 41.95	41.86 ± 32.24	0.471
ALB	38.83 ± 7.98	36.76 ± 6.75	36.44 ± 5.34	0.14
LDH	234.39 ± 156.42	326.23 ± 699.87	226.84 ± 128.55	0.414
PT	12.31 ± 1.67	12.23 ± 1.84	12.52 ± 1.37	0.701
*Liver cirrhosis*				0.231
Absent	44 (66.67)	43 (76.79)	33 (80.50)	
Present	22 (33.33)	13 (23.21)	8 (19.50)	
*Tumor thrombus*				0.777
Absent	41 (62.12)	29 (51.79)	25 (61.00)	
Branch of portal vein	4 (6.06)	3 (5.36)	2 (4.90)	
Main portal vein	21 (31.82)	24 (42.86)	14 (34.1)	
*Extrahepatic metastasis*				0.328
Absent	37 (56.06)	27 (48.21)	17 (41.5)	
Present	29 (43.94)	29 (51.79)	24 (58.5)	
*Smoker*				0.994
Never	48 (72.72)	40 (71.43)	29 (70.70)	
Previous	9 (13.64)	7 (12.50)	6 (14.60)	
Now	9 (13.64)	9 (16.07)	6 (14.60)	
*Alcoholism*				0.403
Never	49 (74.24)	47 (83.93)	34 (82.90)	
Previous	9 (13.64)	6 (10.71)	2 (4.90)	
Now	8 (12.12)	3 (5.36)	5 (12.20)	
*Surgery*				0.653
Yes	18 (27.27)	17 (30.36)	9 (22.00)	
No	48 (72.73)	39 (69.64)	32 (78.00)	
*Radiotherapy*				0.827
Yes	9 (13.64)	6 (10.71)	6 (14.60)	
No	57 (86.36)	50 (89.29)	35 (85.40)	

*HPT* initial hepatic artery intervention combined with immunotarget therapy; *PTH* immunotarget therapy sequential hepatic artery interventional therapy; *PT* immunotarget therapy only

*No (%)

As of May 10, 2022 (data cutoff), the median follow-up time was 18.3 months (95% CI 16.7 to 20.0 months); the cycles of PD-1 inhibitors plus targeted therapy in the HPT group, PTH group, and PT group were 2–29, 2–16, and 2–28, respectively; and the median cycles were 4.5, 5, and 5, respectively. The catalog of PD-1 inhibitors and targeted medicine in each group is shown in Table S2.

### Survival analysis

By the end of follow-up period, 150 of 163 (92.02%) patients had disease progression, including 57 of 66 (86.36%) in the HPT group, 53 of 56 (94.64%) in the PTH group, and 40 of 41 (97.56%) in the PT group.

**Fig. 2:**
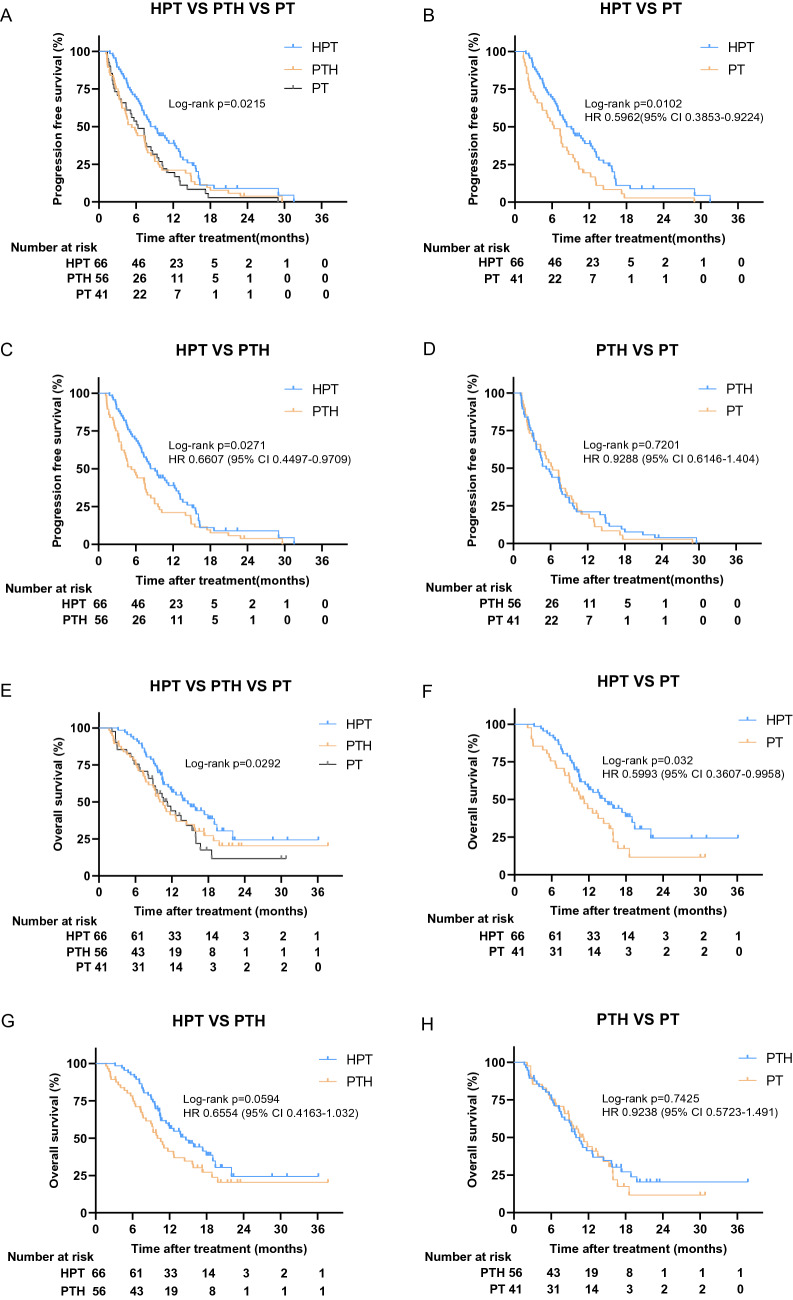
Kaplan–Meier curves of survival outcomes of patients in the three groups. **A** Comparison of progression free survival among three groups. **B** The PFS comparison between HPT and PT (**B**), HPT and PTH (**C**), PTH and PT (**D**). **E** Comparison of overall survival among three groups. The OS comparison between HPT and PT (**F**), HPT and PTH (**G**), PTH and PT (**H**). *HPT* initial hepatic artery intervention combined with immunotarget therapy; *PTH* immunotarget therapy sequential hepatic artery interventional therapy; *PT* immunotarget therapy only

The progression-free survival benefit with cotreatment of targeted therapy (HPT), initial hepatic artery intervention, and PD-1 inhibitors was shown in the three groups (Fig. 2A). An HR of 0.60 (95% CI 0.39–0.92, *p* = 0.01) for the comparison was recorded (Fig. 2B). The median progression-free survival was 8.37 months (95% CI 6.35–10.39) with HPT versus 6.33 months (95% CI 3.75–8.92) with PT. Similar results were observed for HPT versus PTH, with an HR of 0.66 (95% CI 0.45–0.97, *p* = 0.027) for the comparison (Fig. 2C), and the median PFS with PTH was 5.3 months (95% CI 3.48–7.12). However, there was no difference in PFS between the PTH and PT groups (HR 0.93, 95% CI 0.61–1.40, *p* = 0.72) (Fig. 2D). The 6-month and 12-month PFS rates of the HPT group were 69.7% and 34.8%, respectively, which were better than those of the other two groups. The 6-month and 12-month PFS rates in the PTH and PT groups were 44.6% and 53.7% and 19.6% and 17.1%, respectively.

At the time of data cutoff, 110 of 163 (67.5%) patients had died, including 39 of 66 (59.1%) patients in the HPT group, 39 of 56 (69.6%) patients in the PTH group, and 32 of 41 (78.0%) in the PT group. All patients died due to progressive disease.

In the HPT, PTH, and PT groups, 27/66 (40.9%), 17/56 (30.4%) and 9/41 (22%) patients failed to reach OS. According to the current data analysis, the median overall survival was 14.6 months (95% CI 10.6–18.7) in the combined therapy group (HPT) versus 10.0 months (95% CI 8.2–11.8) in the sequential therapy group (PTH) versus 11.3 months (95% CI 8.3–14.3) in the immunotarget therapy group (PT). The 1-year OS rates in the HPT, PTH, and PT groups were 50%, 33.9%, and 34.1%, respectively, while in the 18-month group, OS rates were 21.2%, 14.3%, and 7.3%, respectively. Overall survival in the HTP group showed a survival benefit among the three groups (Fig. 2E). Overall survival was significantly longer in the HTP group than in the PT group with an HR of 0.60 (95% CI 0.361–0.996; *p* = 0.032; Fig. 2F). Compared with the PTH group, the overall survival of the HTP group had a prolonged survival trend with an HR of 0.66 (95% CI 0.416–1.032; *p* = 0.059; Fig. 2G). However, there was no significant difference in overall survival between the PTH and PT groups with an HR of 0.92 (95% CI 0.572–1.491; *p* = 0.743; Fig. 2H).

**Fig. 3:**
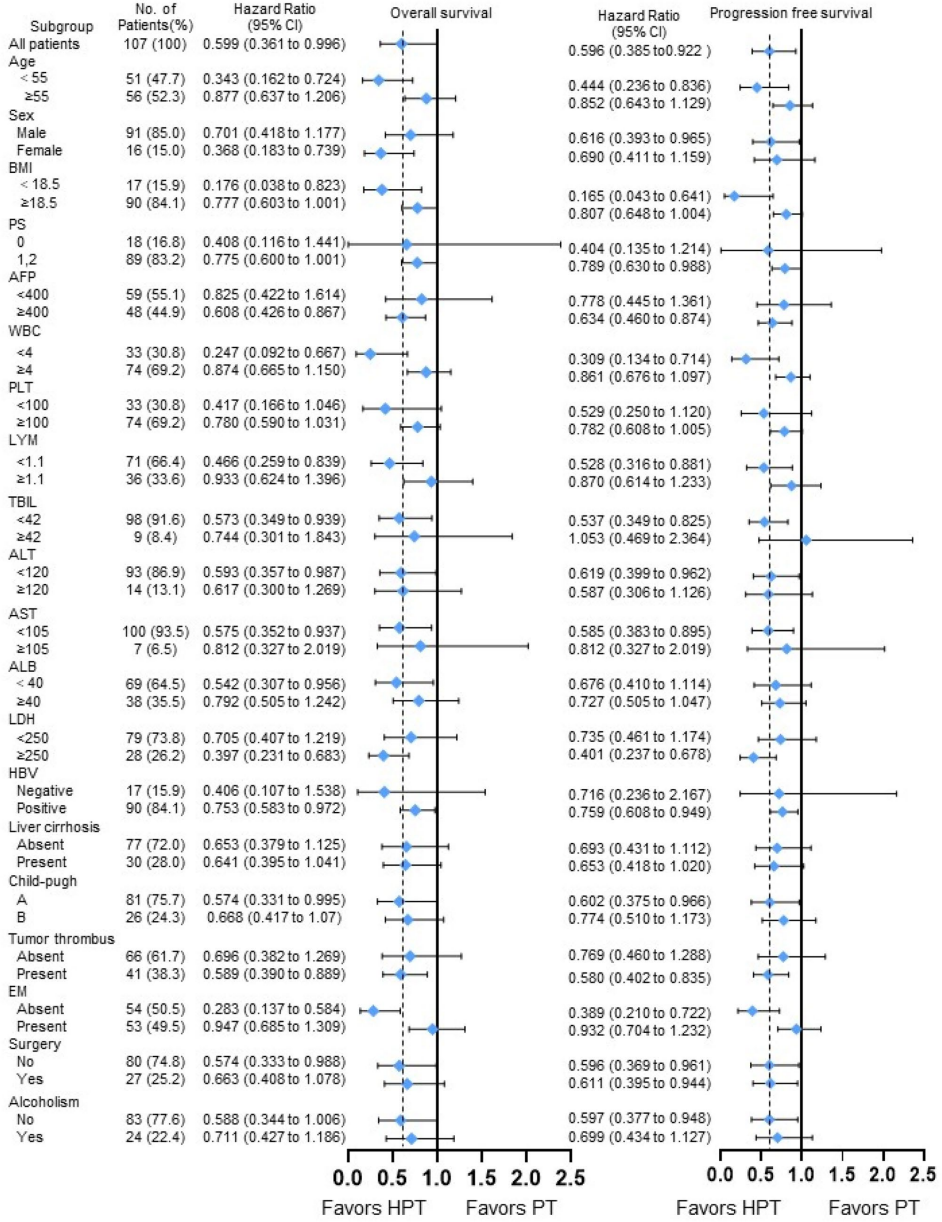
Forest plot for overall survival and progression free survival of the matched cohorts of patients. *HPT* initial hepatic artery intervention combined with immunotarget therapy; *PT* immunotarget therapy only

In pairwise comparisons of the three treatment groups, only the HPT group versus the PT group showed significant differences in overall survival, so subgroup analysis based on the baseline demographics and clinical characteristics of the HPT group and the PT group suggested that HPT was superior to PT in collective patient survival. Subgroup analysis is summarized in Fig. 3.

### Preliminary efficacy

As of May 10, 2022, 2 patients achieved a complete response (CR), and 38/66 (57.6%) achieved a partial response (PR) as their best response in the HPT group, which was significantly better than the PTH and PT groups. The overall response rate (ORR) of the HPT group was 60.6%, which was significantly higher than that of the PTH (32.1%) and PT (22%) groups. The disease control rate (DCR) of the HPT group was 84.8%, which was the highest among the three groups (Table 2).

**Table 2:** Summary of best response

Variable	HPT (*n* = 66) (%)	PTH (*n* = 56) (%)	PT (*n* = 41) (%)	P0* value	P1* value	P2* value	P3* value
Overall response							
Complete response	2 (3.0)	0 (0.0)	0 (0.0)	0.162	0.189	0.261	1
Partial response	38 (57.6)	18 (32.1)	9 (22.0)	< 0.001	0.005	< 0.001	0.269
Stable response	16 (24.2)	24 (42.9)	24 (58.5)	0.002	0.029	< 0.001	0.127
Progressive response	10 (15.2)	14 (25.0)	8 (19.5)	0.394	0.173	0.558	0.524
Overall response rate	40 (60.6)	18 (32.1)	9 (22.0)	< 0.001	0.002	< 0.001	0.269
Disease control rate	56 (84.8)	42 (75.0)	33 (80.5)	0.394	0.173	0.558	0.524

*P0 HPT VS PTH VS PT; *P1 HPT VS PTH; *P2 HPT VS PT; *P3 PTH VS PT

### Tolerability and safety

All patients tolerated treatment, and none of the patients discontinued treatment or died as a result of adverse events. The adverse events with an incidence of more than 10% in all three groups were fatigue and appetite loss. The incidence of gastrointestinal reactions was higher in the hepatic artery intervention combined and sequential therapy groups than in the immunotarget therapy group. The most common grade 3 or 4 adverse events were nausea and appetite loss. There were no significant differences in adverse events among the three treatment groups (Table 3).

**Table 3:** Treatment-related adverse events

Adverse events	Any grade				Grade 3/4			
	HPT (*n* = 66)	PTH (*n* = 56)	PT (*n* = 41)	*P* value	HPT (*n* = 66)	PTH (*n* = 56)	PT (*n* = 41)	*P* value
Treatment-related AEs, *n* (%)								
Rash	5 (7.6)	4 (7.1)	2 (4.9)	0.86	0 (0.0)	0 (0.0)	0 (0.0)	1.00
Pruritus	9 (13.6)	5 (8.9)	4 (9.8)	0.68	0 (0.0)	0 (0.0)	0 (0.0)	1.00
Fatigue	10 (15.2)	9 (16.1)	5 (12.2)	0.86	2 (3.0)	1 (1.8)	0 (0.0)	0.53
Appetite loss	11 (16.7)	9 (16.1)	8 (19.5)	0.89	4 (6.1)	2 (3.6)	2 (4.9)	0.82
Nausea	9 (13.6)	7 (12.5)	1 (2.4)	0.15	5 (7.6)	2 (3.6)	0 (0.0)	0.16
Hypothyroidism	10 (15.2)	5 (8.9)	4 (9.8)	0.51	0 (0.0)	0 (0.0)	0 (0.0)	1.00
Diarrhea	6 (9.1)	4 (7.1)	2 (4.9)	0.72	0 (0.0)	0 (0.0)	0 (0.0)	1.00
Myocarditis	0 (0.0)	0 (0.0)	0 (0.0)	1.00	0 (0.0)	0 (0.0)	0 (0.0)	1.00
Pneumonia	7 (10.6)	5 (8.9)	4 (9.8)	0.95	0 (0.0)	0 (0.0)	0 (0.0)	1.00
Drug-induced liver injury	7 (10.6)	4 (7.1)	2 (4.9)	0.55	2 (3.0)	1 (1.8)	0 (0.0)	0.53
White blood cell count decreased	4 (6.1)	2 (3.6)	1 (2.4)	0.63	2 (3.0)	1 (1.8)	0 (0.0)	0.53
Anemia	3 (4.5)	2 (3.6)	1 (2.4)	0.85	1 (1.5)	1 (1.8)	1 (2.4)	0.94
Platelet count decreased	8 (12.1)	3 (5.4)	2 (4.9)	0.27	3 (4.5)	1 (1.8)	2 (4.9)	0.65
Drug-induced kidney injury	1 (1.5)	1 (1.8)	0 (0.0)	0.71	0 (0.0)	0 (0.0)	0 (0.0)	1.00

### Prognostic factor analysis

Univariate and multivariate prognostic factor analyses for overall survival and progression-free survival are summarized in Table. Multivariate analysis identified that initial hepatic artery intervention combined with immunotarget therapy (HPT), serum albumin level >  = 40, Child‒Pugh A, and hepatectomy history were independent prognostic factors of OS and PFS. In addition, a platelet level >  = 100 was also found to be an independent prognostic factor of PFS but not of OS (Table 4).

**Table 4:** Univariate and multivariate analysis of risk factors for overall survival and progression-free survival

Variables	Overall survival						Progression-free survival					
	Univariate analysis			Multivariate analysis			Univariate analysis			Multivariate analysis		
	HR	95%CI	*P* value	HR	95% CI	*P* value	HR	95% CI	*P* value	HR	95% CI	*P* value
Treatment (HPT/PT)	0.560	0.350–0.897	0.016	0.628	0.462–0.854	0.003	0.600	0.398–0.904	0.015	0.370	0.215–0.637	< 0.001
Age (y), (< / ≥ 55)	0.886	0.553–1.419	0.614				0.973	0.648–1.462	0.897			
Sex, (male/female)	0.970	0.517–1.819	0.923				0.980	0.563–1.706	0.942			
BMI, (< 18.5/ ≥ 18.5)	1.097	0.560–2.149	0.787				1.061	0.576–1.954	0.849			
PS, (0/1–2)	0.880	0.462–1.675	0.697				0.833	0.486–1.429	0.507			
AFP (ng/ml), (< / ≥ 400)	0.581	0.363–0.929	0.023				0.749	0.499–1.124	0.163			
WBC (10^9/L), (< / ≥ 4)	1.541	0.909–2.613	0.108				1.219	0.784–1.895	0.380			
PLT (10^9/L), (< / ≥ 100)	1.395	0.830–2.347	0.209				1.402	0.902–2.179	0.133	2.131	1.084–4.189	0.028
LYM (10^9/L), (< / ≥ 1.1)	1.176	0.715–1.934	0.523				1.245	0.813–1.907	0.314			
TBIL (umol/l), (< / ≥ 42)	0.828	0.357–1.918	0.660				0.672	0.311–1.454	0.313			
ALT (U/L), (< / ≥ 120)	0.576	0.293–1.130	0.109				0.698	0.377–1.291	0.252			
AST (U/L), (< / ≥ 105)	0.572	0.229–1.428	0.232				0.779	0.339–1.790	0.556			
ALB (g/L), (< / ≥ 40)	2.335	1.364–3.997	0.002	2.121	1.165–3.859	0.014	1.961	1.254–3.065	0.003	1.956	1.131–3.383	0.016
LDH (U/L), (< / ≥ 250)	0.781	0.460–1.324	0.358				0.995	0.620–1.598	0.985			
HBV, (no/yes)	1.136	0.596–2.167	0.698				1.259	0.724–2.191	0.415			
Liver cirrhosis, (no/yes)	0.791	0.463–1.352	0.392				0.714	0.452–1.128	0.149			
Child–pugh (A/B)	0.571	0.335–0.974	0.040	0.449	0.232–0.869	0.017	0.664	0.411–1.071	0.093	0.558	0.316–0.985	0.044
Tumor thrombus (no/yes)	1.327	0.814–2.164	0.257				1.169	0.766–1.785	0.470			
EM (no/yes)	0.869	0.545–1.387	0.557				0.764	0.511–1.142	0.190			
Surgery (no/yes)	1.193	0.696–2.045	0.522	1.912	1.006–3.634	0.048	1.608	1.019–2.537	0.041	2.705	1.462–5.004	0.002
Alcoholism (no/yes)	0.839	0.476–1.479	0.543				0.920	0.562–1.506	0.739			

## Discussion

The SHARP trial involved the usage of targeted therapies, and sorafenib demonstrated protracted OS for advanced HCC (Llovet et al. 2008). However, the trial exhibited a restricted impact of anti-PD-1/PD-L1 monotherapy for the treatment of HCC (Kudo 2020). Multiple studies have shown that targeted therapy plus PD-1/PD-L1 inhibitors are superior to targeted therapy alone (Cheng et al. 2022; Ren et al. 2021). Immunotarget therapy with a median PFS of 4.6–6.8 months and ORR of 20–30% still failed to meet clinical needs (Ren et al. 2021). In recent years, encouraging results have been made regarding the efficacy of the triple combination, and the ORR and DCR were approximately 50–70% and 80–100%, respectively (Liu et al. 2021; Yang et al. 2022). Unfortunately, these results were from studies on small sample populations with fewer than 50 participants. The deployment of hepatic artery intervention, PD-1 inhibitors, and targeted medicine has not been reported.

The present retrospective study revealed that initial hepatic artery intervention combined with immunotarget therapy (HPT) had a survival benefit compared to sequential hepatic artery intervention (PTH) and immunotarget therapy alone (PT). The median progression-free survival was 8.37 months (95% CI 6.35–10.39) with HPT versus 5.3 months (95% CI 3.48–7.12) with PTH versus 6.33 months (95% CI 3.75–8.92) with PT. The 1-year OS rates in the HPT, PTH, and PT groups were 50%, 33.9%, and 34.1%, respectively. OS was significantly longer in the HTP group than in the PT group (HR 0.60, 95% CI 0.361–0.996, *p* = 0.032). Compared with the PTH group, the overall survival of the HTP group had a prolonged survival trend (HR 0.66, 95% CI 0.416–1.032, *p* = 0.059). Subgroup analysis suggested that all patients treated with initial hepatic artery intervention combined with immunotarget therapy were superior to those treated with immunotarget therapy alone in terms of OS and PFS. The ORR and DCR of HPT were 60.6% and 84.8%, respectively, which were higher than those of the PTH group and PT group. There was no difference in the safety of treatment modes among the three groups.

Targeted therapies, such as tyrosine kinase inhibitors (TKIs), vascular endothelial growth factor (VEGF) monoclonal antibodies, PD-1 inhibitors, and hepatic artery interventional therapies, have synergistic mechanisms (Li et al. 2021b). HCC is a hypervascular tumor with multiple signaling pathways (Schulze et al. 2015) that easily metastasizes and recurs, and angiogenesis plays a crucial role in tumorigenesis. The antitumor effects of TACE and HAIC are mediated by cytotoxic agents and embolization, resulting in tumor necrosis. Stressed tumor cells compensatively upregulate VEGF and promote angiogenesis (Chang et al. 2020). TKI agents, such as sorafenib and lenvatinib are multitarget VEGF inhibitors that reduce angiogenesis and normalize abnormal vessels, facilitate the entry of cytotoxic agents into the tumor, and achieve higher concentrations. Tumor growth and progression are associated with immune microenvironment inhibition (Huang et al. 2022). Chemotherapeutic agents directly activate immune effects by enhancing antigen cross-presentation, T lymphocyte expansion, and tumor-infiltrating T cells (Shurin et al. 2009; Nowak et al. 2003). Oxaliplatin induced an immunogenic type of cell death in a TLR4/high-mobility group box-1-dependent manner (Obeid et al. 2007; Apetoh et al. 2007). Hepatic artery interventional therapy not only locally eliminates tumors but also activates the host immune system to enhance the antitumor effect of PD-1 inhibitors.

Multivariate analysis suggested that the mode of treatment, albumin level, Child‒Pugh grade, and hepatectomy history were independent prognostic factors for BCLC-C HCC patients. The mechanism and clinical outcome of initial hepatic artery intervention combined with immunotarget therapy suggested that it was the optimal treatment, which had been described previously. The albumin level, Child‒Pugh grade, and availability of hepatectomy reflect liver function reserve, which is correlated with response and survival. A meta-analysis reported that PS >  = 1, AFP > 1,000 ng/ml, extrahepatic spread, and aspartate aminotransferase are independent prognostic values associated with poor outcomes (Llovet et al. 2022); however, these factors did not affect survival in our study and may be related to multiple treatment combinations and therapeutic drugs.

We acknowledge several limitations in this study. First, this was a retrospective study of the diversity of TKI and PD-1 inhibitors resulting in subtle differences in efficacy. Second, the sample was limited, and all of the patients were from Asian populations and thus ethnically homogenous. Third, the follow-up time was insufficient, and OS data were immature because 40.9% of patients in the HPT group did not reach OS.

Despite these limitations, the results demonstrated that the initial hepatic artery intervention combined with an immunotarget therapy pattern could prolong OS and PFS without increasing adverse events for BCLC-C HCC patients. It provides a novel treatment mode for the clinical management of advanced HCC.

## Conclusion

Initial hepatic artery intervention combined with immunotarget therapy gained survival benefits with tolerable side effects compared with immunotarget sequential hepatic artery intervention and immunotarget therapy alone. Multivariate analysis suggested that liver reserve function was closely correlated with prognosis. Which patient populations would benefit most from which treatment mode remains to be further explored in future studies and in clinical practice.

## Supplementary Information

Below is the link to the electronic supplementary material.

Supplementary file1 (DOCX 24 KB)
