# Supplementary material for: Hepatic artery intervention combined with immune-targeted therapy is superior to sequential therapy in BCLC-C hepatocellular carcinoma
Source: J Cancer Res Clin Oncol. 2022 Dec 1;149(8):5405–16. doi: 10.1007/s00432-022-04386-3 (PMC10349705; doi:10.1007/s00432-022-04386-3)
Supplement: Supplementary file 1 — Supplementary file1 (DOCX 24 KB) [file 432_2022_4386_MOESM1_ESM.docx]

Hepatic artery intervention combined with immune-targeted therapy is superior to sequential therapy in BCLC-C hepatocellular carcinoma

Hanzhi Dong^1^, Yan Jian^1^, Zhiqiang Peng^2*^, Meijian Wang^1^, Na Cheng^3*^, Fangfang Liu^4^, Qi Zhang^5^, Wenfeng Zhang^3*^

1. Department of Medical Oncology, Jiangxi Cancer Hospital, The Second Affiliated Hospital of Nanchang Medical College, Jiangxi Clinical Research Center for cancer, Nanchang 330029, China.
2. Department of Lymphohematology, Jiangxi Cancer Hospital, The Second Affiliated Hospital of Nanchang Medical College, Jiangxi Clinical Research Center for cancer, Nanchang 330029, China.
3. Department of Infectious Diseases, The First Affiliated Hospital, Nanchang University, Nanchang 330006, Jiangxi, China.
4. The First Affiliated Hospital, Nanchang University, Nanchang 330006, Jiangxi, China.
5. Department of Liver Disease Center, Shenzhen Hospital of Southern Medical University, Shenzhen 518000, Guangdong, China.

*Corresponding author:

Wenfeng Zhang, Department of Infectious Diseases, The First Affiliated Hospital, Nanchang University, Nanchang 330006, Jiangxi, China.

Email: ndyfy02166@ncu.edu.cn.

Na Cheng, Department of Infectious Diseases, The First Affiliated Hospital, Nanchang University, Nanchang 330006, Jiangxi, China.

Email: chengnah@sina.com

Zhiqiang Peng, Department of Lymphohematology, Jiangxi Cancer Hospital, The Second Affiliated Hospital of Nanchang Medical College, Jiangxi Clinical Research Center for cancer, Nanchang 330029, China. Email: ndzhlyy1277@ncu.edu.cn

Supplement Tables

Table S1. Category and dosage of target medicine and PD-1 inhibitors

| **Category** | **Dose**  **(mg)** | **Category** | **Dose**  **(mg)** |
| --- | --- | --- | --- |
| Sorafenib | 400 bid | Pembrolizumab | 200 |
| (Bayer Schering) |  | (Carlow, Merck Sharp & Dohme Corp) |  |
| Lenvatinib | 8-12 | Toripalimab | 240 |
| (Eisai China Inc) |  | (Suzhou, Hezhong pharmaceutical Co.Ltd) |  |
| Regorafenib | 160 | Sintilimab | 200 |
| (Bayer AG) |  | (Suzhou, Xinda pharmaceutical Co.Ltd)) |  |
| Bevacizumab | 15mg/kg | Camrelizumab | 200 |
| (Roche Pharma Co.Ltd) |  | (Jiangsu Hengrui Medicine Co.Ltd) |  |

**Table S2. Category of target medicine and PD-1 inhibitors in three groups**

| **Category** | **HPT**  **(n=66)** | **PTH**  **(n=56)** | **PT**  **(n=41)** |
| --- | --- | --- | --- |
| Lenvatinib plus Pembrolizumab | 5 (7.6) * | 3 (5.4) | 1 (2.4) |
| Lenvatinib plus Camrelizumab | 30 (45.4) | 29 (51.7) | 21 (51.2) |
| Sorafenib plus Toripalimab | 6 (9.1) | 8 (14.3) | 3 (7.3) |
| Sorafenib plus Sintilimab | 12 (18.2) | 6 (10.7) | 9 (21.9) |
| Regorafenib plus Camrelizumab | 6 (9.1) | 3 (5.4) | 4 (9.8) |
| Bevacizumab plus Camrelizumab | 7 (10.6) | 7 (12.5) | 3 (7.3) |

*No (%)

HPT: initial hepatic artery intervention combined with immunotarget therapy; PTH: immunotarget therapy sequential hepatic artery interventional; PT: PD-1 inhibitors plus target medicine
